# Alpha-CGRP as a specific response mediator during acute myocardial infarction in humans: findings from an observational longitudinal study

**DOI:** 10.3389/fcvm.2025.1581056

**Published:** 2025-06-16

**Authors:** G. Gárate, L. Gangas, J. M. de la Torre, M. Muñoz San-Martín, J. Pascual, V. González-Quintanilla

**Affiliations:** ^1^ Laboratorio de Cefaleas y Otras Enfermedades Neurológicas no Degenerativas, Instituto de Investigación Marqués de Valdecilla (IDIVAL), Santander, Spain; ^2^ Service of Neurology, Hospital Universitario Marqués de Valdecilla, Santander, Spain; ^3^ Departamento de Medicina y Psiquiatría, Universidad de Cantabria, Santander, Spain; ^4^ Service of Cardiology, Hospital Universitario Marqués de Valdecilla, Santander, Spain

**Keywords:** alpha-CGRP, biomarker, myocardial infarction, cardiac cephalgia, response mediator

## Abstract

**Introduction:**

Calcitonin gene-related peptide (CGRP), particularly its alpha isoform, might play a role in restoring physiological cardiovascular functioning. While its involvement in acute myocardial infarction (AMI) pathophysiology has been suggested, human data remain scarce. This study analyzed circulating alpha-CGRP levels during AMI, comparing them to healthy controls (HC) and post-AMI resolution levels.

**Methods:**

A total of 26 AMI patients and 26 age- and sex-matched HC were recruited. Blood samples were collected from patients within four hours of AMI onset and, when possible, six months post-event. Alpha-CGRP serum concentrations were measured using a validated ELISA assay.

**Results:**

Alpha-CGRP levels were significantly higher in AMI patients at admission (mean ± SD: 96.0 ± 77.4 pg/ml) compared to HC (42.0 ± 25.8 pg/ml, *p* < 0.0001), with an average increase of 129%. Among nine patients available for follow-up, levels normalized to the HC range (45.1 ± 26.7 pg/ml, *p* = 0.011). Patients with poor outcomes had numerically lower alpha-CGRP levels (72.6 ± 37.2 pg/ml) than those with a satisfactory resolution (100.3 ± 82.5 70.6 pg/ml; *p* = 0.241).

**Discussion:**

Alpha-CGRP is acutely elevated during AMI, likely as a compensatory vasodilator response to ischemia. Its post-AMI normalization suggests a transient protective mechanism. Further research is needed to explore its role in AMI-related pathophysiology and usefulness as a therapeutic agent.

## 1 Introduction

The calcitonin gene related peptide (CGRP) is a neuropeptide, part of the calcitonin family of peptides. This molecule has two isoforms, alpha and beta-CGRP. These two isoforms are encoded in different genes of the 11th human chromosome and share 34 out of the 37 amino acids of their sequences (1). Regarding their distribution, alpha-CGRP is predominantly expressed in the central and peripheral nervous system, a relevant location for most of the physiological and pathophysiological processes where CGRP may be involved and the reason why this is the isoform which has been more extensively studied, while beta-CGRP is more abundant within the enteric nervous system (2), which has driven its research in relationship with different gastrointestinal conditions (3).

Alpha-CGRP is a multifunctional neuropeptide generated by tissue-specific alternative-splicing of the primary transcript of the calcitonin gene (CALCA) (1). Sensory nerves of the peripheral nervous system, whose cell bodies locate at the dorsal root ganglia, are the prominent sites of alpha-CGRP synthesis. From here, a dense perivascular alpha-CGRP containing neural network originates, terminating around blood vessels in all vascular beds (4).

CGRP has a robust vascular activity, being the most potent microvascular vasodilator known to date (5), producing long-lasting vasodilation that has been described in cerebral, coronary, and kidney vascular beds (6). It has positive chronotropic, inotropic, and pro-hypertrophic effect in humans (7). The alpha isoform, despite its location and effects on the human vasculature, does not play a pivotal role in the physiological control of the blood pressure (1), but it may rather act as a compensatory factor to restore normal cardiovascular functioning in response to pathophysiological challenges such as ischemia, hypertension or heart failure (8).

Although there is some evidence supporting a protective role of alpha-CGRP during an acute myocardial infarction (AMI), most of the data come from animal models (7, 8, 9), and the evidence we have from human studies are still scarce. Following an AMI, increased plasma CGRP levels have been reported which then normalized after its resolution (10) and/or progression (11). Moreover, CGRP infusion during congestive heart failure significantly improves the cardiac function (12) and there is growing evidence that its exogenous administration enhances the recovery after the resolution of these events (13, 14). The mechanisms behind these actions remain unknown, however, it has been suggested to involve vasodilation and generation of protective mediators secondary to CGRP signalling (1).

The beneficial vascular protective effects of alpha-CGRP have driven its research as a potential therapeutic agent beyond its usefulness for the treatment of migraine (15). Nevertheless, the overall clinical weight of the role of alpha-CGRP in mediating a favourable response to the cited events has still to be evaluated with more studies and data.

Here, our aim was to analyse how the circulating concentrations of alpha-CGRP may change during the course of an AMI, either by comparing with controls without any cardiovascular event history (HC), or with the same individuals after AMI resolution.

## 2 Materials and methods

### 2.1 Patients and controls

The study group consisted of patients who had been admitted to our Coronary Care Intervention Unit from 8 am to midday due to confirmed AMI, with and without ST-segment elevation, which had begun less than 4 h earlier. These individuals were recruited from January 2022 to December 2023. Diagnosis of AMI was based on the Fourth Universal Definition of Myocardial Infraction (16).

Parallel to the recruitment of AMI patients we carried out the inclusion of HC, who were paired by sex and age to our AMI group. These individuals were recruited from healthy volunteers at our hospital. Inclusion criteria for the controls were the absence of a history of migraine and/or any other kind of primary headache as well as no history of acute ischemic events nor other active systemic diseases. Additionally, eligible controls could not suffer from obesity, be current smokers, or have been taking any kind of medications continuously for the last 3 months.

The study was conducted according to the guidelines of the Declaration of Helsinki, and approved by the Ethics Committee of Investigations with Medications of Cantabria (28/2020, December 11, 2020). Written informed consent was obtained from all subjects involved in this study.

### 2.2 Sample collection, processing and storage

Blood was extracted from the antecubital vein for all the subjects of the study, always before the endovascular treatment, between 8 am and midday. In the follow-up and control sampling, individuals were on fasting conditions (at least 10 h). After extraction, tubes were left to clot for 10 min, then immediately centrifuged at 3,500 rpm for 10 min to obtain serum, which was transferred into sterile tubes without adding any kind of additional reagent such as protease inhibitors, and stored at −80°C until assayed. In those cases when it was possible, a follow-up sample was obtained 6 months after the resolution of the AMI event. None of the samples remained more than 3 months stored before being analysed.

### 2.3 CGRP determinations

Quantification of alpha-CGRP levels was done using a commercial enzyme-linked immunosorbent assay (ELISA) kit from Abbexa (abx257902), strictly following the protocol set by the manufacturer. This kit employs two different antibodies using the sandwich ELISA technology to specifically detect the alpha isoform of the peptide. The use of this kit has been internally validated by our group, ensuring the reproducibility and reliability of results, with and intra and inter-assay coefficients of variation below 8% and 10%, respectively, and the results of this validations have been published somewhere else (17).

### 2.4 Data analysis

Categorical variables are reported as percentages, whereas continuous variables are displayed as mean ± standard deviation (SD) for the variables which are normally distributed and for those not-normally distributed as mean ± SD along with median and interquartile range (IQR).

For group comparison of categorical variables, the Fisher's exact test was carried out. Normality assumption of the quantitative variables has been checked using the Shapiro–Wilk test. For not-normally distributed data the statistical significance between groups was assessed by the Mann–Whitney *U*-test, while the independent samples *t*-test was used when comparing normally-distributed data. For the evaluation of the differences of alpha-CGRP levels before and after the resolution of the AMI, the Wilcoxon signed-rank test was employed. Correlation between variables was assessed by Spearman Correlation Test.

The *p*-values presented are for two-tailed testing, and to prove statistical significance a *p*-value < 0.05 was considered. All analyses were performed using IBM SPSS Statistics, version 29.0.2.0.

## 3 Results

**Figure 1:**
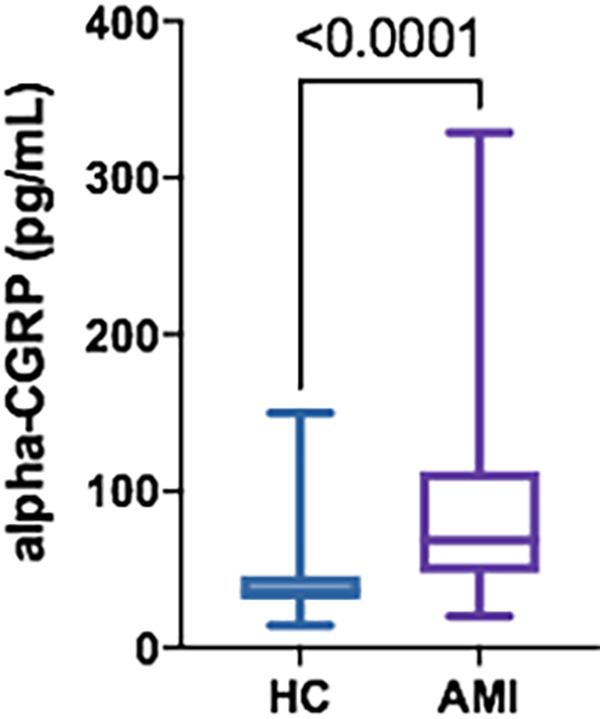
Serum alpha-CGRP levels in HC (*n* = 26) vs. AMI patients (*n* = 26). Data is displayed using Box and Whisker Plot with line at median, boxes showing IQR and bars representing the range.

A total of 26 patients with AMI were recruited (96.2% men; 60.3 ± 10.7 years), along with 26 HC (96.2% men; 61.3 ± 11.3 years). Clinical characteristics of the AMI group are illustrated in Table 1. The distribution of sex and age between groups did not show significant differences (*p* = 0.999; *p* = 0.647; respectively). None of the patients conforming the AMI group had received nitro-glycerine or analgesics before sampling. The AMI group had a significant increase in their alpha-CGRP circulating concentrations at the moment of the AMI [mean ± SD: 96.0 ± 77.4 pg/ml; median [IQR]: 68.8 [49.4–112.9] pg/ml] compared to the HC group [42.0 ± 25.8 pg/ml; 36.3 (31.0–46.0) pg/ml; *p* < 0.0001] (Figure 1), with an average increase of 129%. There were no significant differences in alpha-CGRP circulating concentrations in patients with AMI of the branches of the right coronary artery [95.2 ± 63.6 pg/ml; 70.6 (65.0–105.6) pg/ml] vs. branches of the left coronary artery [96.9 ± 68.5 pg/ml; 68.5 (36.9–123.7) pg/ml; *p* = 0.471]. There were 5 patients who had an unfavourable clinical evolution (death in three cases and severe ischemic myocardiopathy with congestive heart failure in two). Their alpha-CGRP levels were numerically lower [72.6 ± 37.2 pg/ml; 53.2 (42.6–68.5) pg/ml] than those found in patients with a positive outcome [100.3 ± 82.5 70.6 pg/ml; (64.4–110.1) pg/ml; *p* = 0.241]. There was no correlation between admission [20,503 ± 54,426 ng/L; 3,334 (863–20,841) ng/L; *p* = 0.721] or peak [97,837 ± 129,736 ng/L; 29,936 (8,626–133,658) ng/L; *p* = 0.951] troponin levels and serum alpha-CGRP concentrations.

**Table 1:** Main characteristics of AMI patients.

Clinical variable	AMI
Age (years; mean ± SD)	48.9 ± 9.4
Male: *n* (%)	25 (96.2)
Serum CGRP [pg/ml; median (IQR)]	68.8 [49.4–112.9]
Obesity: *n* (%)	4 (15.4)
Current smoker: *n* (%)	8 (30.8)
Arterial hypertension: *n* (%)	11 (42.3)
Type 2 diabetes mellitus: *n* (%)	22 (84.6)
Hypercholesterolemia: *n* (%)	11 (42.3)
Positive evolution: *n* (%)	22 (84.6)
Myocardial Artery Infarcted: *n* (%) Left anterior descending artery Right coronary artery	13 (50) 13 (50)

**Figure 2:**
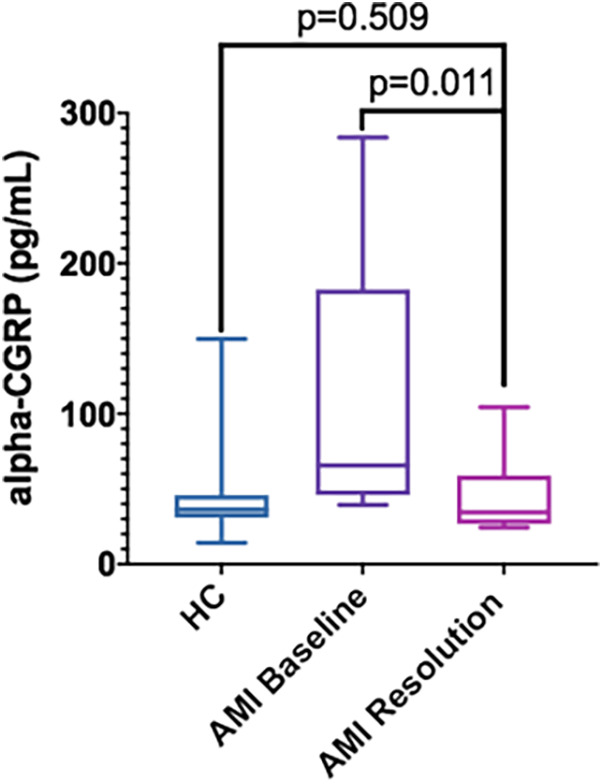
Serum alpha-CGRP levels in HC (*n* = 26) vs. AMI patients at admission (AMI Baseline) (*n* = 9) and at the follow-up visit (AMI Resolution) (*n* = 9). Data is displayed using Box and Whisker Plot with line at median, boxes showing IQR and bars representing the range.

A total of 9 patients (34.6%) attended the follow-up visit and agreed to provide a new blood sample for CGRP determination. Blood samples were not available from the remaining patients mainly because they were not followed-up in our hospital but by their reference cardiologists in other geographical areas. Three patients had died. In this subgroup of patients, we found a normalization of their alpha-CGRP serum concentrations [follow-up: 45.1 ± 26.7 pg/ml; 34.5 (26.7–59.0) pg/ml] whose significant differences compared to the HC disappeared (*p* = 0.509). We also found significant intra-patient differences with an average increase of 149% during AMI [AMI: 112.4 ± 90.6 pg/ml; 65.6 (46.0–182.7) pg/ml; *p* = 0.011] compared to the circulating levels in the follow-up (Figure 2).

## 4 Discussion

Our analysis of the circulating concentrations of alpha-CGRP in patients suffering from an AMI shows a significant increase in the levels of this peptide when compared with a group of HC and with the same individuals six months after the resolution of the event. Moreover, patients with an impaired resolution of the event had lower alpha-CGRP levels during AMI than those with a favourable evolution. These results suggest that CGRP is released as a protective response mediator against the event, trying to counteract the ischemic situation of the myocardium.

As it has been said before, the exact mechanisms by which CGRP mediates a hypothetical protective response are unknown. Our study was not designed to elucidate these mechanisms, but to confirm previous results (8, 10) indicating an upregulation in the expression of CGRP under heart failure conditions. These pre-existing evidences date from the 1990s decade and, despite their conceptual value, these studies were performed in a date where intervention protocols for AMI were different: while in these works patients were treated with thrombolytic agents, our patients underwent a thrombectomy followed by a stenting procedure. Also, there was limited knowledge about the specificity of technique employed for CGRP measurement, whether they were measuring alpha or beta-CGRP, and regarding how to develop a working protocol for CGRP measuring in blood samples that ensure the validity and reproducibility of results. Now that our knowledge about CGRP has expanded and methodological studies have been published (17, 18), we wanted to evaluate the specificity of increased levels of alpha-CGRP for AMI and analyse its biological implications with a proven, validated and published protocol (17) in patients suffering AMI sampled right before the stenting procedure. In any case our results are both consistent and complementary to those priorly published.

CGRP is mostly known for being a new therapeutic target for migraine (19). Alpha-CGRP in particular has also been postulated as the first non-clinical biomarker for chronic migraine (20, 21). However, and besides the data here presented about alpha-CGRP concentrations in AMI, several conditions and diseases other than chronic migraine have been linked to altered levels CGRP, such as inflammatory bowel diseases (22, 23), COVID-19 infections (24), or periodontal inflammation (25). The implications of this redundancy has proved a limited specificity of increased levels of serum alpha-CGRP circulating concentrations, and these data in AMI serve as a further example. Furthermore, it is noteworthy to highlight that the elevation found in this study is, to the best of our knowledge, the biggest found across the conditions where CGRP is altered, with an average increase of 129% compared to HC. Moreover, intra-patient comparison during the AMI and after its resolution supports the specificity of the increase, given that the levels normalize to the range here described in HC and show an average increase of 149% compared to regular conditions. Taking all together, it is probably that the alteration of alpha-CGRP here described is produced as a consequence of AMI itself and not to other factors known to alter the circulating concentrations of CGRP, such as age/sex differences or presence of comorbidities, as these were carefully considered in our analysis to avoid the confounding effect of these variables.

In our work, and as it happens with all the studies analysing circulating concentrations of the molecule, the source of CGRP release is unknown. After synthesis, alpha-CGRP is stored in large vesicles within the sensory nerves innervating the heart. These nerves are sensitive to ischemic, constricted and cytotoxic environments (26), which makes them the most likely source of CGRP in the subjects suffering from an AMI. Therefore, the release can be produced directly to the heart, where it would carry out its protective function, either by vascular-dependent and myocardium vascular-independents pathways (8). It is from this primary location where alpha-CGRP would reach our sample source, the peripheral circulation, where alpha-CGRP could act on other peripheral targets.

Interestingly, there is a secondary migraine-like headache known as cardiac cephalgia, which was first described in 1997 by Lipton and colleagues (27), included in the *International Classification of Headache Disorders* (ICHD-3) (28), and whose prevalence is situated around 6% of the cases of AMI (29). The pathophysiology for this headache remains to be demonstrated, although different hypotheses have been raised (30). Our results support the proposal of an induction of the headache caused by the effect of biochemical mediators released by and/or within the heart muscle, more specifically alpha-CGRP. The causative role of this molecule in migraine pain is well-known (1), and, based on this information, we could hypothesize that the release of alpha-CGRP into the peripheral circulation could reach the trigeminovascular system, where it would start a signalling pathway that ultimately would induce the cardiac cephalalgia. Whether this alpha-CGRP release does or does not result in the development of the headache would depend on the concentration of the molecule in the peripheral circulation and on the individual level of sensitization of the trigemino-vascular system, whose activation is responsible of migraine attacks. We did not collect information regarding the presence of cardiac cephalgia, but it leaves the opportunity to further test our hypothesis in future studies.

Our study has some limitations. First, we could not follow-up all the patients initially included in our work and, even though the differences between AMI and its resolution show a robust normalization, the sample size is rather small. On the other hand, we did not collect data regarding the possible presence of cardiac cephalgia, which would have been an interesting data to analyse in combination with our results. As a consequence, there is still room for further explore our results, with bigger sample sizes and a more detailed *ad-*hoc clinical data collection.

Overall, our results show that alpha-CGRP is acutely elevated during AMI, likely as a compensatory response to ischemia, and its normalization after the resolution of the event suggests a transient mechanism. Further research is needed to explore its role in AMI-related pathophysiology and its possible usefulness as a therapeutic agent.

## Data Availability

The raw data supporting the conclusions of this article will be made available by the authors, without undue reservation.

## References

[1] Russell FA King R Smillie SJ Kodji X Brain SD. Calcitonin gene-related peptide: physiology and pathophysiology. Physiol Rev. (2014) 94(4):1099–142. 10.1152/physrev.00034.2013 25287861 PMC4187032

[2] Mulderry PK Ghatei MA Spokes RA Jones PM Pierson AM Hamid QA Differential expression of α-CGRP and β-CGRP by primary sensory neurons and enteric autonomic neurons of the rat. Neuroscience. (1988) 25(1):195–205. 10.1016/0306-4522(88)90018-8 2839796

[3] Russo AF Hay DL. CGRP Physiology, pharmacology, and therapeutic targets: migraine and beyond. Physiol Rev. (2023) 103(2):1565–644. 10.1152/physrev.00059.2021 36454715 PMC9988538

[4] Uddman R Edvinsson L Ekblad E Håkanson R Sundler F. Calcitonin gene-related peptide (CGRP): perivascular distribution and vasodilatory effects. Regul Pept. (1986) 15(1):1–23. 10.1016/0167-0115(86)90071-6 3532219

[5] Brain SD Williams TJ Tippins JR Morris HR MacIntyre I. Calcitonin gene-related peptide is a potent vasodilator. Nature. (1985) 313(5997):54–6. 10.1038/313054a0 3917554

[6] Brain SD Grant AD. Vascular actions of calcitonin gene-related peptide and adrenomedullin. Physiol Rev. (2004) 84(3):903–34. 10.1152/physrev.00037.2003 15269340

[7] Kumar A Potts JD DiPette DJ. Protective role of α-calcitonin gene-related peptide in cardiovascular diseases. Front Physiol. (2019) 10:821. 10.3389/fphys.2019.00821 31312143 PMC6614340

[8] Argunhan F Brain SD. The vascular-dependent and -independent actions of calcitonin gene-related peptide in cardiovascular disease. Front Physiol. (2022) 13:833645. 10.3389/fphys.2022.833645 35283798 PMC8914086

[9] Li J Levick SP DiPette DJ Janicki JS Supowit SC. Alpha-calcitonin gene-related peptide is protective against pressure overload-induced heart failure. Regul Pept. (2013) 185:20–8. 10.1016/j.regpep.2013.06.008 23816470

[10] Mair J Lechleitner P Längle T Wiedermann C Dienstl F Saria A. Plasma CGRP in acute myocardial infarction. Lancet. (1990) 335(8682):168. 10.1016/0140-6736(90)90040-c 1967457

[11] Lechleitner P Genser N Mair J Dienstl A Haring C Wiedermann CJ Calcitonin gene-related peptide in patients with and without early reperfusion after acute myocardial infarction. Am Heart J. (1992) 124(6):1433–9. 10.1016/0002-8703(92)90054-y 1462896

[12] Aubdool AA Thakore P Argunhan F Smillie SJ Schnelle M Srivastava S A novel *α*-calcitonin gene-related peptide analogue protects against end-organ damage in experimental hypertension, cardiac hypertrophy, and heart failure. Circulation. (2017 Jul 25) 136(4):367–83. 10.1161/CIRCULATIONAHA.117.028388 28446517 PMC5519346

[13] Gennari C Nami R Agnusdei D Fischer JA. Improved cardiac performance with human calcitonin gene related peptide in patients with congestive heart failure. Cardiovasc Res. (1990) 24(3):239–41. 10.1093/cvr/24.3.239 2346957

[14] Bentsen S Sams A Hasbak P Edvinsson L Kjaer A Ripa RS. Myocardial perfusion recovery induced by an α-calcitonin gene-related peptide analogue. J Nucl Cardiol. (2022) 29(5):2090–9. 10.1007/s12350-021-02678-8 34089154 PMC9553834

[15] Kumar A Williamson M Hess A DiPette DJ Potts JD. Alpha-Calcitonin gene related peptide: new therapeutic strategies for the treatment and prevention of cardiovascular disease and migraine. Front Physiol. (2022) 13:826122. 10.3389/fphys.2022.826122 35222088 PMC8874280

[16] Thygesen K Alpert JS Jaffe AS Chaitman BR Bax JJ Morrow DA Fourth universal definition of myocardial infarction (2018). Circulation. (2018) 138(20):e618–51. 10.1161/CIR.0000000000000617 30571511

[17] Gárate G Pascual J Pascual-Mato M Madera J Muñoz-San Martín M González-Quintanilla V. Untangling the mess of CGRP levels as a migraine biomarker: an in-depth literature review and analysis of our experimental experience. J Headache Pain. (2024) 25(1):69. 10.1186/s10194-024-01769-4 38684990 PMC11057141

[18] Messlinger K Vogler B Kuhn A Sertel-Nakajima J Frank F Broessner G. CGRP Measurements in human plasma—a methodological study. Cephalalgia. (2021) 41(13):1359–73. 10.1177/03331024211024161 34266288 PMC8592105

[19] Edvinsson L Haanes KA Warfvinge K Krause DN. CGRP As the target of new migraine therapies—successful translation from bench to clinic. Nat Rev Neurol. (2018) 14(6):338–50. 10.1038/s41582-018-0003-1 29691490

[20] Cernuda-Morollón E Larrosa D Ramón C Vega J Martínez-Camblor P Pascual J. Interictal increase of CGRP levels in peripheral blood as a biomarker for chronic migraine. Neurology. (2013) 81(14):1191–6. 10.1212/WNL.0b013e3182a6cb72 23975872

[21] Gárate G González-Quintanilla V González A Pascual M Pérez-Pereda S Madera J Serum alpha and Beta-CGRP levels in chronic migraine patients before and after monoclonal antibodies against CGRP or its receptor. Ann Neurol. (2023) 94(2):285–94. 10.1002/ana.26658 37038806

[22] Pascual-Mato M Gárate G González-Quintanilla V Madera-Fernández J Castro B García MJ Differences in circulating alpha-calcitonin gene-related peptide levels in inflammatory bowel disease and its relation to migraine comorbidity: a cross-sectional study. Headache. (2024) 64(7):849–58. 10.1111/head.14768 38922858

[23] Pascual-Mato M Gárate G González-Quintanilla V Castro B García MJ Crespo J Unravelling the role of beta-CGRP in inflammatory bowel disease and its potential role in gastrointestinal homeostasis. BMC Gastroenterol. (2024) 24(1):262. 10.1186/s12876-024-03366-w 39134940 PMC11320777

[24] Gárate G Pascual M Rivero M Toriello M Pérez-Pereda S González-Quintanilla V Serum calcitonin gene-related peptide α and β levels are increased in COVID-19 inpatients. Arch Med Res. (2023) 54(1):56–63. 10.1016/j.arcmed.2022.12.002 36588002 PMC9801185

[25] Leira Y Ameijeira P Domínguez C López-Arias E Ávila-Gómez P Pérez-Mato M Periodontal inflammation is related to increased serum calcitonin gene-related peptide levels in patients with chronic migraine. J Periodontol. (2019) 90(10):1088–95. 10.1002/JPER.19-0051 31070784

[26] Preibisz JJ. Calcitonin gene-related peptide and regulation of human cardiovascular homeostasis. Am J Hypertens. (1993) 6(5 Pt 1):434–50. 10.1093/ajh/6.5.434 8390269

[27] Lipton RB Lowenkopf T Bajwa ZH Leckie RS Ribeiro S Newman LC Cardiac cephalgia: a treatable form of exertional headache. Neurology. (1997) 49(3):813–6. 10.1212/wnl.49.3.813 9305346

[28] Xx X. Headache classification committee of the international headache society (IHS). the international classification of headache disorders, 3rd edition. Cephalalgia. (2018) 38(1):1–211. 10.1177/0333102417738202 29368949

[29] Culić V Mirić D Eterović D. Correlation between symptomatology and site of acute myocardial infarction. Int J Cardiol. (2001) 77(2-3):163–8. 10.1016/s0167-5273(00)00414-9 11182180

[30] Ruiz Ortiz M Bermejo Guerrero L Martínez Porqueras R González de la Aleja J. Cardiac cephalgia: when myocardial ischaemia reaches the neurologist’s consultation. Neurologia (Engl Ed). (2020) 35(8):614–5. 10.1016/j.nrl.2019.09.003 31780317

